# Sixty-Seven Years of Land-Use Change in Southern Costa Rica

**DOI:** 10.1371/journal.pone.0143554

**Published:** 2015-11-23

**Authors:** Rakan A. Zahawi, Guillermo Duran, Urs Kormann

**Affiliations:** 1 Las Cruces Biological Station, Organization for Tropical Studies, Apartado 73–8257, San Vito de Coto Brus, Costa Rica; 2 Centro de Recursos Hídricos para Centroamérica y el Caribe, Universidad Nacional de Costa Rica, Sede Chorotega, Liberia, Costa Rica; 3 Department of Agroecology, University of Goettingen, Grisebachstr. 6, D-37077, Goettingen, Germany; Centre for Cellular and Molecular Biology, INDIA

## Abstract

Habitat loss and fragmentation of forests are among the biggest threats to biodiversity and associated ecosystem services in tropical landscapes. We use the vicinity of the Las Cruces Biological Station in southern Costa Rica as a regional case study to document seven decades of land-use change in one of the most intensively studied sites in the Neotropics. Though the premontane wet forest was largely intact in 1947, a wave of immigration in 1952 initiated rapid changes over a short period. Overall forest cover was reduced during each time interval analyzed (1947–1960, 1960–1980, 1980–1997, 1997–2014), although the vast majority of forest loss (>90%) occurred during the first two time intervals (1947–1960, 1960–1980) with an annual deforestation rate of 2.14% and 3.86%, respectively. The rate dropped to <2% thereafter and has been offset by forest recovery in fallow areas more recently, but overall forest cover has continued to decline. Approximately 27.9% of the study area is forested currently. Concomitantly, the region shifted from a single contiguous forest to a series of progressively smaller forest fragments with each successive survey. A strong reduction in the amount of core habitat was paralleled by an increased proportion of edge habitat, due to the irregular shape of many forest fragments. Structural connectivity, however, remains high, with an expansive network of >100 km of linear strips of vegetation within a 3 km radius of the station, which may facilitate landscape-level movement for some species. Despite the extent of forest loss, a substantial number of regional landscape-level studies over the past two decades have demonstrated the persistence of many groups of organisms such as birds and mammals. Nonetheless, the continued decline in the quantity and quality of remaining habitat (~30% of remaining forest is secondary), as well as the threat of an extinction debt (or time lag in species loss), may result in the extirpation of additional species if more proactive conservation measures are not taken to reverse current trends–a pattern that reflects many other tropical regions the world over.

## Introduction

Forested land is being cleared across the globe, leading not only to the loss of habitat and forest fragmentation, but subsequent detrimental effects on biodiversity, associated ecosystem functioning, and climate change e.g., [1–4]. Primarily driven by the rising demand for agricultural and forest products [5–7], this process is particularly pronounced in tropical landscapes, which are the biodiversity strongholds of the world [8]. Some studies estimate that up to 36% of tropical and subtropical forests that are present today could disappear by 2050 [9], with inherent impacts to biodiversity [10].

Although there are detailed indices for loss of forest cover at both the global and country level e.g., [11–13], few studies examine land use conversion at finer spatial scales (e.g., <1000 km^2^) and over longer time spans, but see [14, 15]. This is surprising given that the functionality of remaining habitat–including landscape-scale forest cover, patch size, and connectivity–are critical for understanding small-scale shifts in patterns of biodiversity, and determining the provision of ecosystem services in human-altered landscapes at the local level. Most such studies conclude that forest cover is essential for the maintenance of biodiversity e.g., [16–20], and with extensive field sampling predictive models can be developed for groups such as birds that are based on the amount of remaining forest in a given area [21].

Determining change in land use over time is especially important in heavily researched areas where it can serve to bolster the results of projects that have been conducted. For example, the Las Cruces Biological Station (LCBS) in southern Costa Rica, where this study is focused, forms part of the Mesoamerican biodiversity hotspot [8] and has been a hub for tropical research for over 50 years with more than 900 attributed publications. The LCBS reserve is one of the largest forest fragments in the area protecting ~365 ha of habitat, most of which is classified as primary forest. Many researchers work on a landscape-level scale in the vicinity of the reserve, where changes in the land use of the area have directly impacted the type of studies undertaken and skewed the majority of research to a more applied focus. For example, studies have documented depauperate species communities in small forest fragments [20, 22, 23]; impacts of forest fragmentation on fauna [19, 20, 24]; shifts in herpetofauna composition relative to climate change [25]; and the impacts of fragmentation and isolation on the genetic population dynamics of trees [26]. Most of these studies have restricted their analyses to the impacts of current forest cover on biodiversity parameters, largely ignoring the importance of the legacy effect of previous forest configurations [27] in part because such data were lacking. This could prove deceptive as extinction debts, or the gradual loss of biodiversity as a result of deforestation, can take several decades or longer to transpire [28]. Furthermore, despite the demonstrated capacity for forests to recover in this region after persistent pre-columbian disturbance [29, 30], the extent of clearing in recent times is unprecedented. Accordingly, the long-term stability of remaining forested habitats in these areas is unclear making it essential to quantify recent historical parameters that can help evaluate how prior and present forest cover may impact biodiversity in the future.

Here we quantify a regional case study of land use change over a sixty-seven year period (1947–2014) using aerial photographs and satellite images taken of Coto Brus county in southern Costa Rica. Colonization of this part of Costa Rica occurred relatively late (1940s) and the region soon faced an increasing influx of migrants in the mid 1950s with the settlement of post-WWII Italian refugees in the area [31]. To promote settlement by the Italians, the Costa Rican government set aside 10,000 ha for colonization, which required clearing of substantial tracts of forest habitat. The development led to a boom in the county’s population growth over the next decade, that resulted in most land being converted from forest to shade-grown coffee production, and most farmers became heavily dependent on coffee agriculture for subsistence [32, 33]. Clearing of forest was also encouraged as a practice in Costa Rica, as in many other parts of tropical America, as a means to lay initial ownership claims to a property [34].

Our goal in this study is to characterize the historical and concurrent forest cover in the regional area surrounding LCBS over this sixty-seven year period. In particular, we: (1) assess the change in forest cover by comparing forest attributes over time (age, number, and size of forest patches); and (2) characterize historical and current forest landscapes using a suite of derived parameters (forest structural connectivity, amount of forest edge, and amount of interior or core forest) to generate a historical forest context for this part of Costa Rica that is critical to furthering our understanding of biodiversity patterns and shifts in the present and future.

## Materials and Methods

### Study area

**Fig 1:**
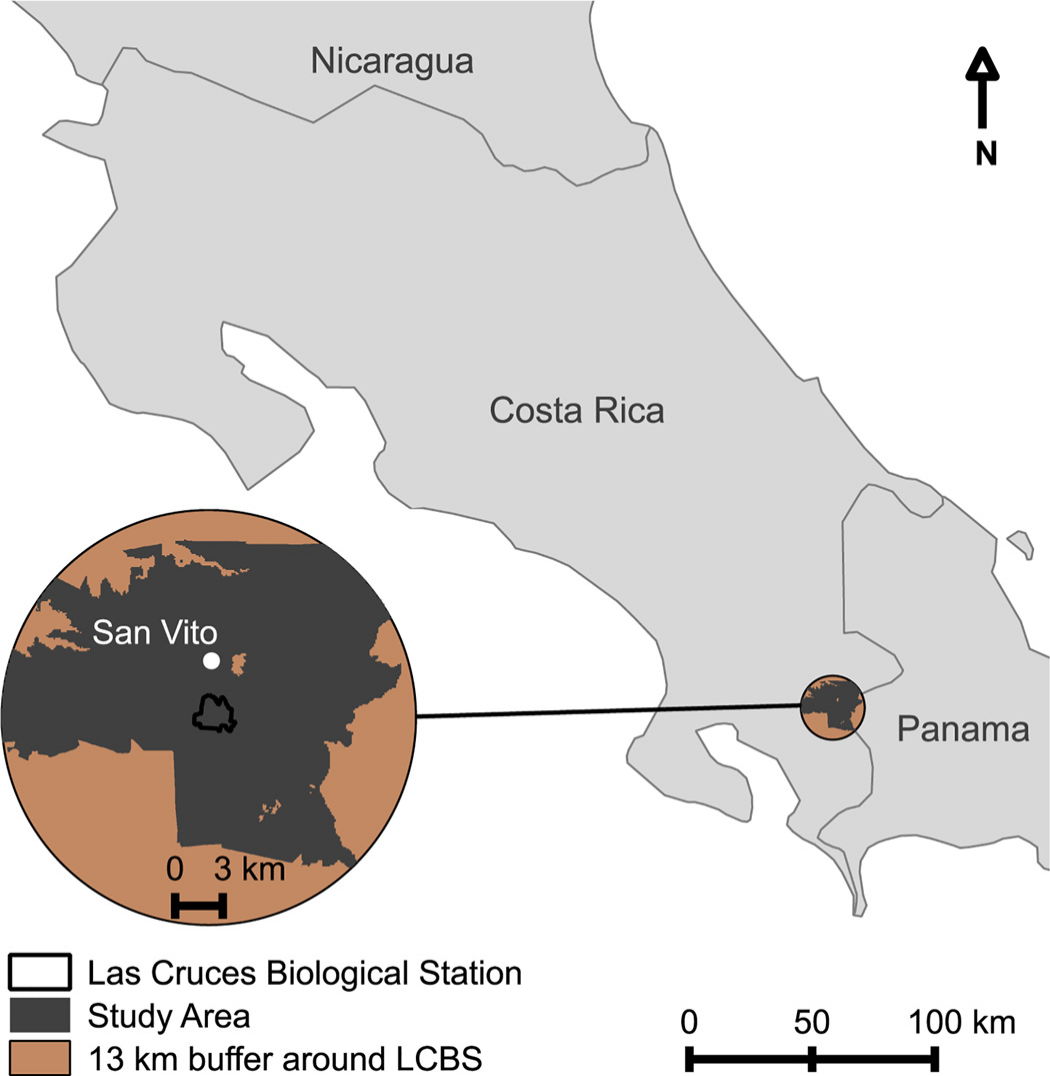
Map of Costa Rica indicating the location of the study area. Areas highlighted in brown were excluded from analyses because they were below the 700 m elevation cutoff, or were located in neighboring Panama.

The study area encompasses a 13 km radius centered at the Las Cruces Biological Station (LCBS; 8° 47' 7” N; 82° 57' 32” W) in Coto Brus county, southern Costa Rica (Fig 1). The area ranges in elevation from ~100–1500 m a.s.l. (based on the Shuttle Radar Topography Mission digital elevation model), however, an elevation cutoff of >700 m.a.s.l. was selected as lowland forest has been subjected to different historical selection pressures [31] than the mid-elevation habitat surrounding the field station. Land to the east of the field station that is on the Panamanian side of the border (~8 km away) was also excluded, as it would also be subject to the selective pressures and historic events of that country (Fig 1). The remaining area, amounting to a total of 32,076 ha (320 km^2^), is classified as a tropical premontane wet forest zone [35], and receives a mean annual rainfall of 3.5–4 m with a pronounced dry season from December to March. Mean annual temperature at LCBS is ~21°C. No permission was required to conduct this study as all information was obtained from purchased/open access aerial images and/or satellite flyovers. No endangered or protected species were involved in this study.

### Images and orthorectification

Five time slices were analyzed based on the available set of aerial imagery: 1947 (B&W), 1960 (B&W), 1980 (B&W), 1997 (true color) and a set of high-resolution Google images taken in 2014 (Table 1). When last accessed (September 2015), Google imagery had 8.0% of the study area covered with lower resolution images. This region falls entirely under the Ngöbe indigenous reserve at the western edge of the study area, a remote and rugged area with no road access and where forest cover has not changed significantly during the period of this study. Accordingly, these areas were replaced with orthophotos taken in 2005; all analyses were conducted on this combined set of images, which we hereafter refer to as 2014.

**Table 1:** Source and relevant information associated with the images used in analyses.

Set of images	Source	Additional information
1947	Images scanned and provided by IGN archive.	Aerial photographs taken by USAF in 1947 (majority) and 1948.
1960	Images scanned and provided by IGN archive.	Aerial photographs taken in March 1960 by USAF at 30,000 feet.
1980	Images scanned and provided by IGN archive.	Aerial photographs taken in January 1980 at 6,000 feet.
1997	Misión TERRA aerial photographs.	Aerial photographs taken between November 1997 and January 1999 by Hauts Monts Inc. for MINAE/RECOPE; scale 1:40,000. Orthorectified using Google Earth imagery, IGN 1:50,000 and CENIGA 1:25,000 cartographic sheets.
2005	Misión CARTA imagery.	Orthophotographs; scale 1:5,000. Accessed through SNIT WMS.
2014	Google Earth high resolution imagery.	Satellite imagery taken 31 December 2013, 1 February 2014*, and 31 March 2014; accessed May 2014 and September 2015. Images by CNES/Astrium.

* Most of the area of interest was captured on this date

CENIGA: Centro Nacional de Información Geoambiental (Costa Rica)

CNES: Centre National d’Etudes Spatiales (France)

IGN: Instituto Geográfico Nacional (Costa Rica)

MINAE: Ministerio de Ambiente y Energía (Costa Rica)

RECOPE: Refinadora Costarricense de Petróleo (Costa Rica)

SNIT: Sistema Nacional de Información Territorial (Costa Rica)

USAF: United States Air Force

WMS: Web Mapping Service

Aerial photographs for the years 1947, 1960, 1980 and 1997 were acquired from the Organization for Tropical Studies GIS Lab and the Instituto Geográfico Nacional of Costa Rica. The orthorectification process was done first on the 1997 set of images and used the current 1:50,000 and 1:25,000 Costa Rican cartography to identify geographical reference points. The set of 1997 orthophotos was used as a reference set to orthorectify remaining years with the exception of 1947 images. The orthorectification process and all other geospatial analyses were done on the CRTM05 spatial reference system and the resulting orthophotos had a 2m cell size. The largest Root Mean Square error (RMSE) of the orthorectification of these three time slices of aerial photographs was 15 m. Orthophotos of 2005 were accessed through the Centro Nacional de Información Territorial Web Map Service of the Costa Rican government and were displayed together with Google imagery on QGIS (version 2; http://qgis.osgeo.org) using the OpenLayers plugin.

Given the lack of information on flight parameters, and the expansive forest coverage in 1947 photographs, images were georeferenced and built into a mosaic using river basins and the few forest clearings that had a similar shape in the 1960 flyover. The 1947 set of images did not cover the whole study area, having empty areas without photographs that represented ˜12.1% of the analysis extent. However, these areas could be classified as forested given that forest was present in these same areas in the 1960 imagery, there was no historical record of deforestation prior to the 1947–1960 time interval, and there is little likelihood that the area was deforested and then reverted given the general land-use change pattern for the region at that time.

### Forest mapping and landscape analysis

Forest mapping was done by visual interpretation of orthophotos and Google imagery. The areas were considered forested if tree crowns were easily identified when viewing the images at a scale of 1:10,000. In areas where it was difficult to discern the type of land cover, a scale of 1:5,000 was used. This was done to eliminate agroforestry systems such as shaded coffee areas (with trees planted in rows) or very early stages of forest regeneration from the forest land-cover class. The analysis was done only in areas that were cloud free in the five time slices. This resulted in the elimination of 134 ha (~0.4%) from of the original area outlined above. Polygons were drawn over the different areas using QGIS and were transformed into raster files of 10 m cell size. Landscape analyses (patch area, patch size, core area analyses) were carried out using Fragstats 4.2 (http://www.umass.edu/landeco/research/fragstats/fragstats.html) and an 8 cell neighborhood rule; the calculations of forest class and patch metrics were done across the entire study area.

### Deforestation and forest regeneration rate

For all four observation periods (1947–1960, 1960–1980, 1980–1997, 1997–2014), we calculated the annual deforestation rate (ADR) using the equation:

$$ADR=100*(\sqrt[{n}]{\frac{{\text{A}}_{\text{t}+1}}{{\text{A}}_{\text{t}}}}\;-1)$$

*where* A_t_ is the overall forest area at the beginning of a time period; A_t+1_ is the overall forested area, excluding regeneration, at the end of a given time period; n is the duration of the period in years, and ADR the mean annual deforestation rate measured as a percentage. For the 2005/2014 time slice we used 2014 to determine the number of years as most of the study area (92%) was covered by this set of imagery.

Some forest regeneration occurred in the study area during the latter three time intervals. Accordingly we determined the annual reforestation rate (ARR) for three observation periods (1960–1980, 1980–1997, 1997–2014), defined as the amount of forest that has grown on cleared land during a given time period (t to t+1). We used the same equation as above to calculate ARR, using the overall deforested area at the beginning of a time period as A_t_, and A_t_ minus the newly reforested area at the end of the time period as A_t+1_.

### Structural connectivity analysis

Using a 3 km buffer around the perimeter of the Las Cruces reserve as a subsample (~5867.25 ha total area analyzed), all live fences [36], small riverine corridors, and other tree covered areas (those that were not previously considered forest due to their small size) were mapped to determine fragment connectivity using the 2014 Google imagery, and viewed at a scale of 1:5,000 sensu [37], (S1 Fig). For larger tree-covered areas such as shaded coffee systems or early stages of forest regeneration with a few tree canopies present, a straight line was drawn through the middle of the area; if an area was connected to other strips of vegetation the lines were considered to branch from the middle. The total length of all lines or strips of vegetation was determined, as well as the total length of strips that connect any two or more forest patches, strips that are connected to a single forest patch, and strips that are completely isolated.

## Results

### Landscape change

**Fig 2:**
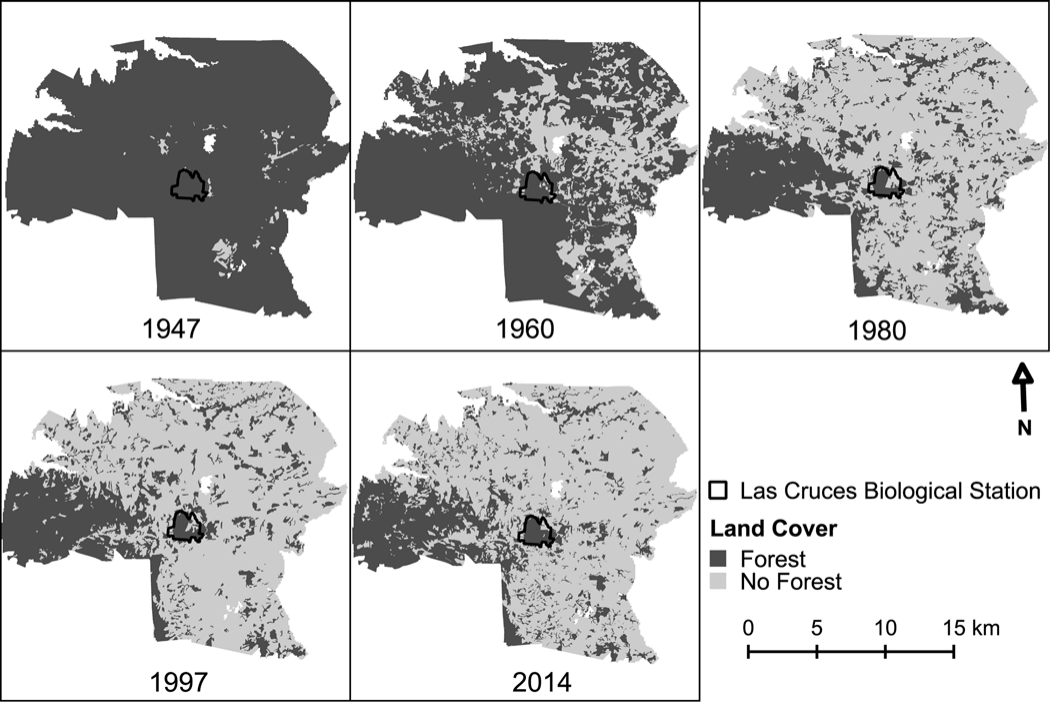
Land use change at five time slices showing extent of forested and non-forested habitat in a 13 km radius around the Las Cruces Biological Station. Land <700 m in elevation was excluded from the analysis (SW region, largely), as was land that fell on the Panamanian side of the border (East).

Total forest cover in the study region declined by 71.6% from ~31,489 ha in 1947, to ~8,951 ha in 2014; roughly 27.9% of the study area is forested at present (Table 2, Fig 2). Most deforestation occurred between 1960–1980 with an ADR of 3.86% (Fig 2); the rate was lower during the first time interval evaluated (2.14%). Although deforestation continued to occur, the rate slowed considerably during the later 1980–1997 period (ADR = 1.06%) but increased in 1997–2014 with an ADR = 1.89%. Almost all forest cover loss (>90%) occurred during the first two time intervals (Table 2).

**Table 2:** Changes in various forest patch metrics during the sixty seven-year time period of the study.

	1947	1960	1980	1997	2014
Forest cover (ha)	31,489	23,761	10,925	9,901	8,951
Forest cover (%)	98.2	74.1	34.1	30.9	27.9
Total length of edge (km)	104.13	1033.34	1233.20	1184.45	1435.88
Area-weighted mean distance between patches (m)	N/A	22.93	46.79	44.60	53.28
Area-weighted mean of patch size (m^2^)	N/A	N/A	2,342	2,245	1,915

N/A–Not Applicable.

Due to the unbroken expanse of forested area in these years, certain calculations were not possible.

**Fig 3:**
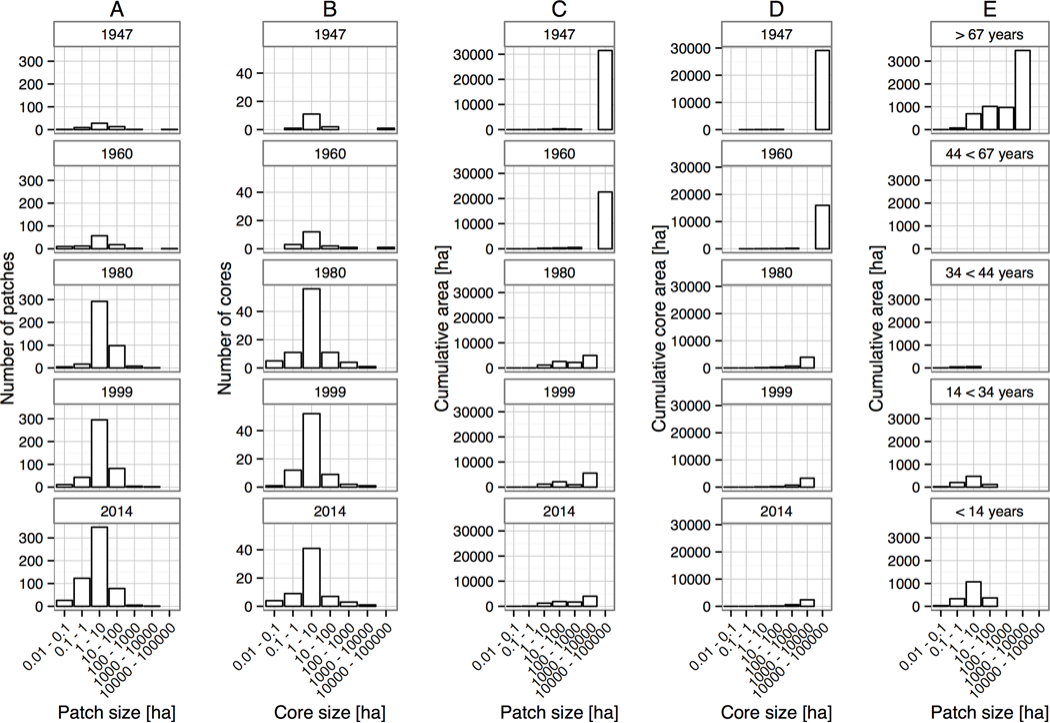
Land use change parameters over five time slices arranged by size class. Column (A) number of patches of forest in each size class; (B) number of core patches (using a 100 m edge buffer) in each size class; (C) cumulative forest area grouped by size class; (D) cumulative core forest area (using a 100 m edge buffer) grouped by size class; and (E) age classification of forest patches present in 2014 and grouped by size class.

**Fig 4:**
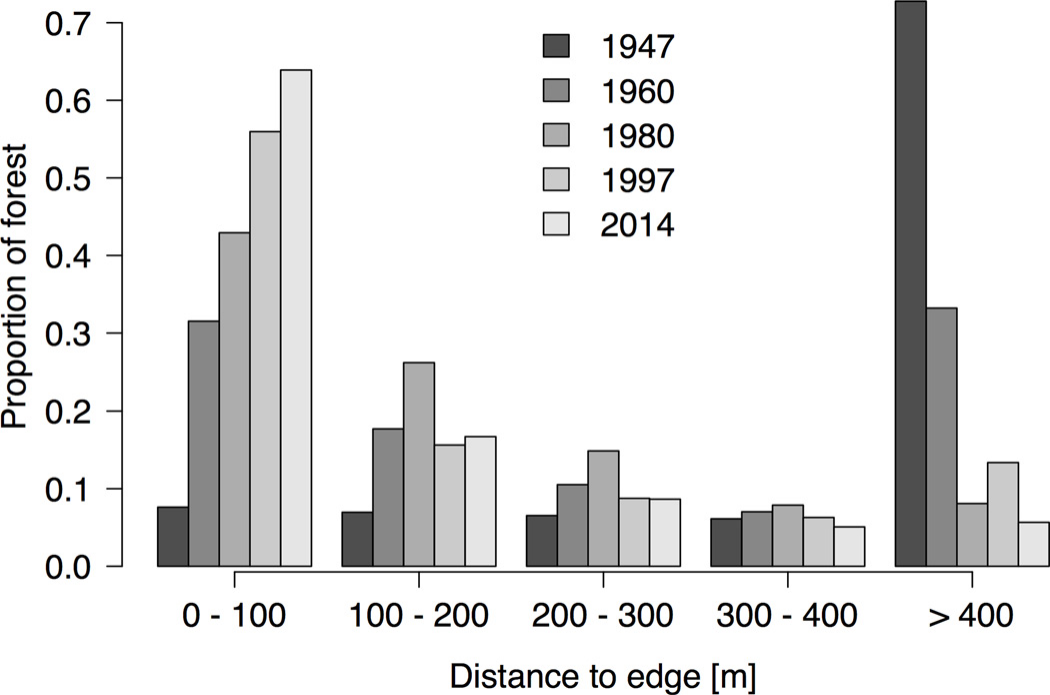
Proportion of forest area remaining grouped by year and distance to nearest forest edge.

Concomitantly, forest patch size shifted from a large single area (1947, and to a lesser extent 1960), to a number of progressively smaller forest patches (Fig 2). The majority of these patches are in the 0.1–1 and 1–10 ha size classes today (Fig 3A). Notable remaining patches include the Ngöbe indigenous reserve (western portion of the study area) and the LCBS reserve. Fragmentation continued to occur after 1980, but it was on a considerably lesser scale and fewer shifts in the categorization of patch sizes are notable (Fig 3A), although the area-weighted mean of patch size continued to decline (Table 2). Similarly, core areas of forest habitat (using a 100 m edge impact criteria) declined over time, from a singular large area to progressively smaller patches (Fig 2, Fig 3B). Strikingly, the number of core forest patches (Fig 3B) is far fewer than the total number of forest patches (Fig 3A) as many are irregular and consequently fall into smaller size classes, or are entirely dropped from the census as they lack a core area. Viewed alternately, the proximity of forest habitat to an edge was essentially inverted between 1947 and 2014 with more than two thirds of remaining forest habitat today located <100 m from a forest edge (Fig 4).

Cumulative area of forest habitat and core forest area habitat (using the same 100 m distance to edge criteria) show similar patterns over time (Fig 3C and 3D). However, the cumulative amount of core area (Fig 3C) as compared to overall forest area (Fig 3D) is substantially lower from 1960 onward. A marked reduction in the amount of habitat present in large areas is notable between 1960 and 1980, as well as a sharp reduction in overall forested habitat area. Not surprisingly, the amount of edge habitat increased significantly from a little over 100 km in 1947 to more than 1,000 km in 1960 (Table 2). Although edge habitat continued to increase in subsequent time periods, increments were more moderated. Nonetheless, the moderate increase in edge habitat between 1960 and 1980 masks the ‘inversion’ of habitat from a primarily forested region (1960) to a highly fragmented one (1980) where the predominant habitat in the landscape is now agricultural land (Fig 2).

### Forest regeneration

**Fig 5:**
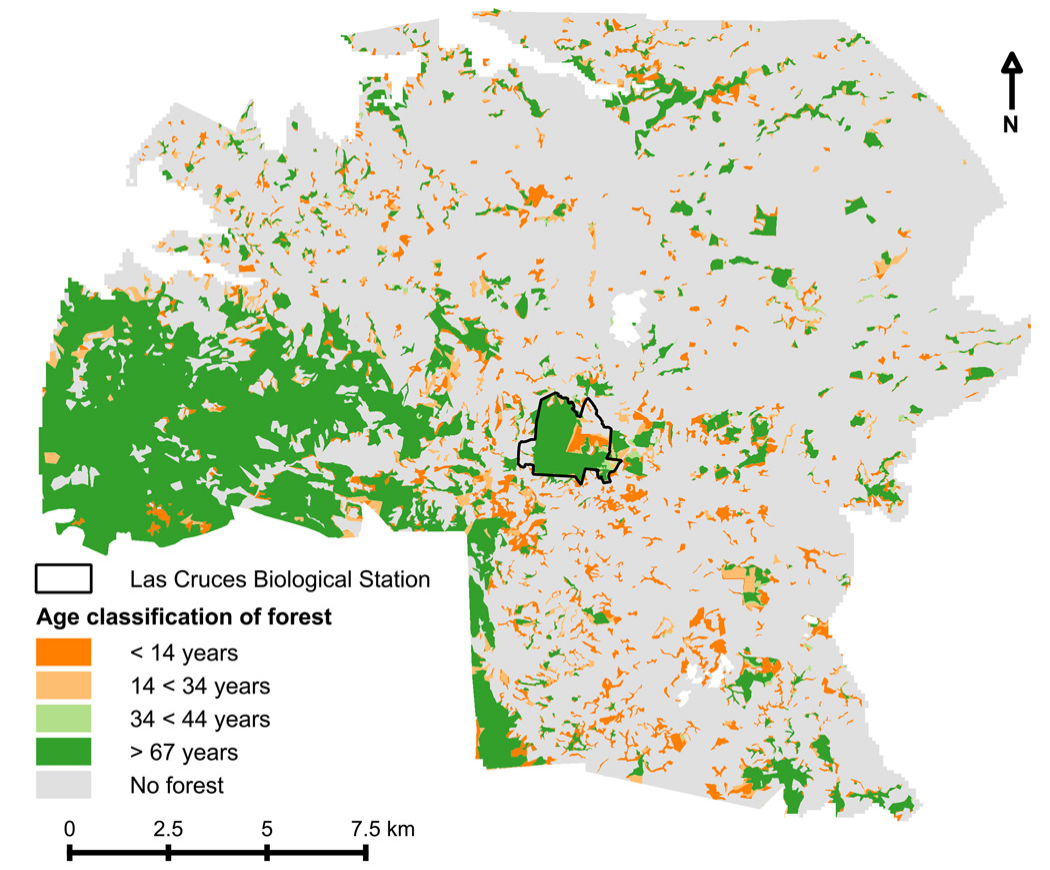
Extant secondary forest cover in 2014 classified by age. Old growth forest (> 67 years) is also depicted. Note that there was no evidence of forest regeneration during the 1947–1960 time interval; accordingly, there are only three forest regeneration classifications.

Most of the extant forest cover (8,951 ha) is old growth that was present before 1947 (Fig 3E). However, some new forest regenerated during the latter three time intervals and helped offset overall forest loss (Fig 5). Overall forest cover loss was offset by 602 ha of secondary growth during 1960–1980 with an annual reforestation rate (ARR) of 0.38%, by 2,186 ha from 1980–1999 (ARR = 0.60%) and by 1,802 ha (ARR = 0.47%) for the most recent time interval. The increase in new forest habitat in the latter two sampling periods also created some larger patches of secondary forest (10–100 ha size class; Fig 3E). Whereas secondary forest accounted for only 5.5% of overall forest cover in 1980, by 2014 this proportion had increased to fully 30.5% (2,731 ha; Fig 5). As of 2014, old growth forest covered only 19.4% of its historical expanse.

### Structural connectivity

The mean distance between forest patches in the study area doubled between 1960 and 2014 (Table 2). Despite the increasing degree of isolation, and the extent of forest fragmentation, a network of 503 linear strips of vegetation was quantified in the 3 km radius surrounding LCBS in the 2014 survey totaling 102,099 linear meters. Of these strips, 19.8% connected two, or more, patches of forest to each other and 45.3% were attached to one patch of forest. Of the 155 forest patches found in this subsampled area, fifty-three (34.1%) were interconnected by linear strips of vegetation.

## Discussion

The analysis of a seven-decade long trajectory revealed that approximately four fifths of the original forest cover has been lost during this time period. Most loss occurred during the first three decades. We characterized the historical and current forest landscapes and documented not only a 10-fold increase in the number of forest fragments, but also strong shifts towards smaller size classes with most of the remaining forest habitat impacted by edge effects. Overall forest loss has been offset by natural regeneration, particularly in the most recent time period; however, fully 30% of remaining habitat is now classified as secondary forest.

Habitat loss and fragmentation are consistently associated with a decline in biodiversity and the provision of ecosystem services [28, 38]. Despite the scale of forest loss in the study area, however, there is a perhaps surprisingly high degree of biodiversity present for a number of different groups e.g., [17, 39–41]. A key factor in maintaining this diversity is the presence of the Las Cruces reserve. For example, avian mark and recapture studies have shown that forest species have a higher probability of survival in the LCBS fragment as compared to smaller forest fragments [19, 22], and communities in smaller fragments are less stable, due to higher colonization levels, which maintain smaller fragments in a constant state of flux [42]. Some frugivores make additional use of the matrix surrounding LCBS for foraging, although to do so, species spend a much greater proportion of their time in the remaining old-growth trees that dot the landscape [43]. Others maintain a genetically diverse population by using of the network of forest fragments, as demonstrated for the White-ruffed Manakin [44]. Nonetheless, loss of habitat, and conversion to agriculture does lead to a decline in phylogenetic diversity among birds overall–and this is particularly exacerbated under high-intensity agriculture [16].

We found a high degree of structural connectivity in the immediate area surrounding the Las Cruces reserve, with more that 100 km of fencerows and riparian strips in a 3 km radius. This landscape feature helps maintain regional biodiversity, providing connectivity for birds [45, 46], and expands the foraging range for many species e.g., [47]. Many other studies in tropical regions have demonstrated similar results for vertebrates e.g., [48–50], but also for beetles [51]. That said, the current distance weighted mean of ~53 m between forest fragments is substantial for some species, given for example that many forest understory birds rarely cross clearings >30 m even with some measure of connectivity [52]. Using extensive field mist net sampling data in our region, researchers have been able to develop models that can estimate abundance and richness of bird fauna based on such precise land-cover variables [21, 53]. Other fauna, such as reptiles and amphibians, also benefit from this expanded network–increasing their effective habitat by more than an order of magnitude [54]. Nonetheless, two-thirds of forest fragments in this sub-survey were isolated, leaving room for improvement or an opportunity for increased connectivity through additional fencerow plantings e.g., [37, 49].

Several studies in this area have examined the implications of forest fragmentation with respect to the impact on ecosystem services. Karp et al. [55] demonstrated how birds can increase the yield of agricultural crops by reducing infestation of pests–and showed that this effect was stronger in farms that had some degree of adjacent forest habitat. In turn, Hadley and Betts [45] showed that the movement of a hummingbird species across this fragmented landscape was restricted to riparian strips–a finding that has strong implications for plant pollination and seed set [20]. Brosi et al. [56] examined the spatial ecology of bees, and although no difference was found in diversity or abundance across different habitat types, a striking difference was found in composition, with dominance by the alien honeybee *Apis mellifera* in pastures and social stingless bees adjacent to forest patches. Such disparities could have long-term implications for the pollination success of plants in these habitats, with a corresponding impact on bee populations in the area. Interestingly, Euglossine bee abundance is positively related with increasing fragment size, and richness and abundance are positively associated with the amount of fragment edge–likely due to their highly mobility among fragments [23].

### Land-use change in southern Costa Rica

During the time frame considered, the study region lost almost three quarters of its forested habitat. Not only was there wide-scale land conversion and habitat loss, but remaining forested areas were reduced to progressively smaller patches and the predominance of secondary forest increased. This pattern occurred in many other parts of the country [13, 57], but is not unique to Costa Rica–for example, Cayuela et al. [14] documented land-use change in the highlands of Chiapas over a 25 year period and noted an almost 50% decrease in forest cover along with a concomitant ~200% increase in the number of forest fragments in the area. They also noted a major decrease in the size of patches, from a primarily singular large fragment to progressively smaller ones.

Most deforestation in our study area occurred during the first two time intervals examined (1947–1960, 1960–1980); the latter interval coincides with the peak wave of deforestation that occurred in Costa Rica during the 1970s [58, 59]. Deforestation in Costa Rica during this peak period was geared primarily towards the establishment of cattle pastures. It was driven by a series of complex factors including population growth, international market economics (especially beef production), and settlement encouragement through government incentives [57–60]. In Coto Brus, however, most of the early deforestation (1950s and 1960s) was geared toward coffee production or simply to help secure land tenure claims, rather than beef production, in part due to the excellent potential for coffee cultivation and high yields compared to the rest of the country [34], but also due to the degree of isolation from the rest of the country and poor road infrastructure [31]. Although coffee was the principal agricultural commodity for the county for the most of the latter half of the 20^th^ century, land under pasture cultivation continued to increase in the 1970s and 1980s as access to markets improved [31, 61].

Active measures to bring deforestation under control on a national level increased in the late 1970s and 1980s with the establishment of the national parks system, a series of forestry laws designed to regulate and discourage tree felling, and the cessation of pro-squatter laws; changes in market economics for the country also contributed [58, 62]. Although these new laws may have impacted deforestation rates in Coto Brus, forest loss continued to occur but on a somewhat more reduced scale, as most forest had already been cleared by the time they came into effect.

The failure to renew the International Coffee Agreement in 1989 led the collapse of the coffee market in the early 1990s, and Coto Brus was one of the areas to suffer the most [32, 33]. Farmers that remained switched to other forms of agriculture, particularly cattle production as this required minimal financial investment, but also to other crops. Unfortunately high annual rainfall coupled with steep slopes do not lend themselves to cattle production and result in considerable soil degradation and loss of topsoil as has occurred in the older pastures in the study area [63].

Forest regeneration helped offset deforestation values, especially during the most recent time interval. Most of the recent recovery occurred in fallowed coffee fields [32]. Some former agricultural lands were also left to recover once secured in reserves (e.g., land acquisitions by LCBS and other private landowners) and was incentivized by strong changes in forest policy law in the 1990s, which affected recovery in other parts of Costa Rica as well [62]. Interestingly, overall deforestation outpaced reforestation in all time intervals evaluated, even though the province of Puntarenas (to which the study area belongs) registered a net increase in forest cover for the last two decades [62]. Indeed, Costa Rica saw a net increase in reforestation of moist forest during the same time period and the highest such increase for Central America [64]. Future predictions of land use are, nonetheless, hard to discern given the complex interactions between local and distant drivers of land-use change in an ever more connected economy [65].

### Evaluating the impact of current land-use distribution on biodiversity

The continued fragmentation of remaining habitat and the increased area of edge habitat can lead to a decline in species richness and composition of trees e.g., [66], reduce sexual reproduction in animal-pollinated plants [44], and even impact the phylogenetic diversity of remaining individuals along a forest edge [67]. At Las Cruces, fragmentation has skewed the phylogenetic composition of future tree generations of *Symphonia globulifera* due to the dominance of a few isolated reproductive individuals, creating a potential regional genetic bottleneck [26]; similar results of inbreeding in other tree species have been documented in other studies in Costa Rica [68]. Shifts in tree community composition have also been noted at Las Cruces and may be driven by subtle changes in climate or long-term edge effects (Zahawi unpublished data). Most remaining habitat in the study area is <100 m from a forest edge, a condition that applies to more than 20% of the worlds’ forests today [69] and a cause for major concern as most edge effects operate in this range [70]. Tree mortality is particularly elevated up to 100 m from an edge [71], with strong negative effects up to 300 m for large canopy trees (>60 cm DBH) [72] that provide important ecological services such as fruit production and carbon storage.

Despite the resilience of most regional fauna to the sharp reduction in forest habitat, there are considerable concerns for the region. First, the shift in land use toward greater reliance on pastoral agriculture [32] could reduce the degree of habitat permeability for forest-dependent species to move between forest patches. Numerous studies have shown that pasture is a particularly poor habitat for fauna e.g., [73] and most animals actively avoid entering such areas. Furthermore, almost one third of remaining habitat is now classified as secondary forest, which does not support the same species composition as that found in mature forest. Second, whereas resilience of species has been shown to be strong, the threat of an extinction debt for most species is far greater when regional forest cover is reduced to less than 30% [74], as is the case in this study area. This effect is likely exacerbated for species that are particularly sensitive to edge habitat and rely on greatly reduced core areas due to the irregular shape of forest fragments [75]. Indeed, some evidence for an extinction debt in this area is already emerging (Betts unpublished data), reflecting results found for similar habitats in other regions [1]. Third, subtle changes in climate are also causing distinct shifts in community composition, as demonstrated in a 42-year resurvey of frogs in the Las Cruces reserve [25]. Such shifts are of particular concern in fragmented landscapes where the ability of certain groups of fauna to migrate to more favorable environments is impeded by habitat discontinuity.

Although reversing all the current threats to biodiversity and ecosystem services in the area may not be possible, assisting in the recovery of strategic habitat areas, either through active restoration or passive natural regeneration, would help offset the threat of extinction debts for different groups of organisms. Furthermore, continued protection and buffering of the larger forest fragments that are scattered in this landscape matrix is essential, as most studies have demonstrated the disproportionate role that they play in the conservation of regional biodiversity.

## Supporting Information

S1 Fig Structural connectivity in a 3 km radius around the Las Cruces Biological Station. (EPS)
